# Recruitment, Retention, and Training of Citizen Scientists in Translational Medicine Research: A Citizen Science Initiative on Non-Alcoholic Fatty Liver Disease

**DOI:** 10.7759/cureus.56038

**Published:** 2024-03-12

**Authors:** Syed Ghulam Sarwar Shah, Yolanda Barrado-Martín, Thomas Marjot, Jeremy W Tomlinson, Vasiliki Kiparoglou

**Affiliations:** 1 Public Health, Oxford University Hospitals National Health Services (NHS) Foundation Trust, Oxford, GBR; 2 Primary Care, University College London, London, GBR; 3 Diabetes and Endocrinology, University of Oxford, Oxford, GBR; 4 Biomedical Sciences, The Griffin Institute, Harrow, GBR

**Keywords:** co-research with members of the public, incentives to citizen scientists, health research, public involvement in research, research and innovation, citizen scientists, biomedical research, metabolic endocrinology, translational medicine research, citizen science

## Abstract

Citizen science is a participatory science approach in which members of the public (citizens) collaborate with scientists and professional researchers and become involved in research and innovation activities, resulting in the co-creation of scientific knowledge and innovation. Citizen science has been widely applied in research, particularly in the social sciences, environmental sciences, information and communication technologies, and public health. However, the application of this approach in clinical sciences, particularly in translational medicine research, is still nascent. This exploratory study involved members of the public (citizen scientists) in a translational medicine experiment on non-alcoholic fatty liver disease that incorporated a lifestyle and weight-loss intervention. The aim of this paper is to report successful methods and approaches for the recruitment, retention, and training of citizen scientists. For the citizen scientists’ recruitment, online calls placed on the websites of our research project and biomedical research center and targeted emails were the most helpful. Of the 14 members of the public who expressed interest in our study, six were recruited as citizen scientists. Citizen scientists were mostly female (n = 5, 83%), white (n = 3, 50%), over 50 years of age (n = 4, 67%), educated to postgraduate level (n = 5, 83%), and either retired or not in employment (n = 5, 83%). The retention rate was 83% (n = 5), and the dropout rate was 17% (n = 1). We arranged instructor-led interactive online training sessions (an hour-long one-on-one session and two-hour group sessions). Research skills training covered ethics in research and qualitative and quantitative data analysis. Citizen scientists were given several incentives, such as reimbursement of travel and care costs, selection as citizen scientists of the month, publications of their blogs and perspective articles, and co-authorship and acknowledgement in papers and project deliverables. To conclude, members of the public (particularly middle-aged white women with postgraduate education) are interested in becoming citizen scientists in translational medicine research. Their retention rate is higher, and they can contribute to different research activities. However, they need training to develop their research skills and expertise. The training should be simple, comprehensive, and flexible to accommodate the schedules of individual citizen scientists. They deserve incentives as they work on a voluntary basis.

## Introduction

Over the last few decades, citizen science approaches to research have been used in different disciplines, mainly in the environment and ecology [1], social and natural sciences [2], and information and communication technology [3]. Literature shows citizen scientists involvement in research has several benefits, such as democratization of science, co-creation of scientific knowledge and innovation, empowerment, and social learning, and increased scientific literacy [4-6]. However, the application of citizen science approaches in the domain of health is underexploited [7] and limited to public health [8,9] because of the ease of involving local communities in public health issues [10]. The interest is increasing in expanding the use of citizen science approaches in other areas of health [11] to reap its benefits, such as exploitation of citizens experiences with health problems, increasing awareness about health issues, and improving health literacy. In addition, some research funders, such as the European Union and the National Institute for Health and Care Research in the United Kingdom, especially call for patient and public involvement and engagement in research, including clinical and biomedical research, particularly in the domain of translational medicine research, where citizen science is still in its infancy [12]. The reasons may include a range of challenges [4], which start from the design of citizen science studies [13] through to the recruitment [14,15], retention [16,17], and training of citizen scientists [17-19]. Earlier studies report that the recruitment of citizen scientists is a cumbersome and resource-intensive activity [13], and despite a lot of planning, effort, and time spent, the success of recruiting citizen scientists and retaining them until the end of a project or study is a major challenge [14]. Similarly, developing citizen scientists’ research skills and expertise in various research activities, as well as understanding of the research process, are also important issues that need special attention because these could improve accuracy and confidence in research [20]. There is, however, a gap in the literature as to which methods and channels are effective in successful recruitment and retention of citizen scientists and in minimizing their dropout of a research project or study, and what are citizen scientists' training needs and preferences to develop their research expertise and skills to maximize their contributions to the research studies, especially in the field of translational medicine research.

This exploratory study applied a citizen science approach in the field of endocrinology and metabolism by conducting a translational medicine experiment on non-alcoholic fatty liver disease that involved a lifestyle and weight loss intervention. The methodological details of this study are published elsewhere [21].

The aim of this paper is to describe successful methods and approaches for the recruitment, retention, and training of citizen scientists and the incentives provided to them. The objectives are to report how we recruited citizen scientists, which were the most successful recruitment methods, what were the retention and dropout rates, which incentives were offered to and exploited by them, what were the research skills development training needs of citizen scientists, and how and what training was provided to them.

## Materials and methods

Study context

This study is part of an interdisciplinary citizen science project called the STEP CHANGE Project, which is a multi-country and multi-partner project funded by the European Commission [28]. The project involves five citizen science initiatives (CSIs) in three distinct but interrelated fields, i.e., health, energy, and environment. The project includes two CSIs on health, one of which is the CSI on NAFLD conducted in the United Kingdom. NAFLD is a metabolic disorder that affects about 25% of the global population [22]. In NAFLD, there is an excessive accumulation of triacylglycerol within the epithelial cells of the liver, which can progress to inflammation, cirrhosis, and liver cancer [23]. Currently, there are no licensed medications for treating NAFLD, and management revolves around lifestyle and weight-loss interventions [24]. Literature shows that lipid metabolism, notably in the liver, is regulated by a circadian rhythm [25]; however, much of the data is based on rodent models [26], and detailed studies in humans are lacking.

The aim of this translational medicine experiment was to study morning and evening (diurnal) variations in the liver fat metabolism in overweight individuals in different conditions (i.e., with and without NAFLD) before and after a lifestyle and weight loss intervention [21]. Commercial providers provided the lifestyle and weight loss intervention for 12 weeks in community settings [27]. Citizen scientists (members of the public) were involved as co-researchers alongside scientific researchers (both clinical and non-clinical).

Selection criteria for citizen scientists

For the recruitment of citizen scientists, we used several selection criteria, i.e., being a member of the public, age 18 or more, and having access to a computer, laptop, or smartphone and the Internet. Citizens of any gender, ethnicity, sexual orientation, or employment status were welcome to participate in the study.

Sampling method and sample size

We recruited citizen scientists using non-random sampling techniques, applying convenience [27] and snowball sampling methods [16]. There is no recommended sample size for recruiting citizen scientists [9], as it depends on the type and context of the research study and the research activities undertaken by citizen scientists. In this exploratory experimental medicine study, we did not fix a specific sample size but decided to at least have parity between the citizen scientists and the professional researchers involved in the study.

Recruitment of citizen scientists

**Figure 1:**
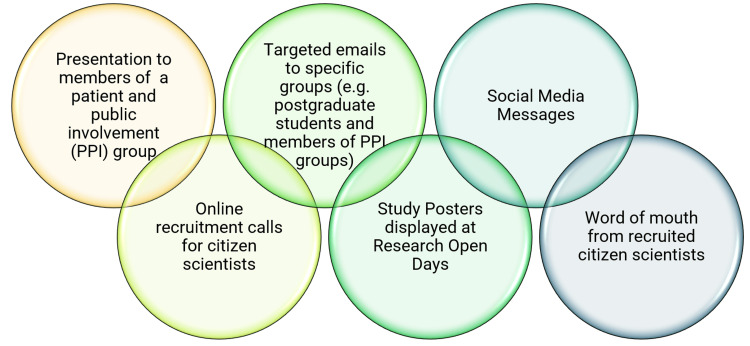
Channels used for the recruitment of citizen scientists.

We used different channels to reach out to the potential citizen scientists (Figure 1). The process for the recruitment started with an online call for citizen scientists’ recruitment, which was placed on the websites of the STEP CHANGE project and the National Institute for Health and Care Research’s (NIHR) Oxford Biomedical Research Centre (BRC). We sent targeted emails to the members of patient and public involvement (PPI) groups associated with our BRC as well as to post-graduate students in the Medical Sciences Division of the University of Oxford. Additional recruitment methods were posters displayed at a research open day of the NIHR Oxford BRC, messages placed on a social media platform, i.e., Twitter (now called X), and word of mouth by the recruited citizen scientists. 

Data collection from citizen scientists

We collected data from citizen scientists as follows:

Expression of Interest

We used a simple form for collecting data from the potential citizen scientists about their expression of interest (EoI) in being involved as citizen scientists in our study. The EoI form asked for their socio-demographic information (i.e., gender, age, and ethnic background), previous research experience (if any), and why they were interested in becoming citizen scientists, what types of research tasks they would like to be involved in, whether they had any caring responsibilities, and their contact details (e-mail address and telephone or mobile phone number). There was no response deadline, but we asked them to complete the EoI form as soon as possible if they wanted to participate in the study. The EoI data was collected through emails sent separately to each potential citizen scientist.

Training Needs

Anticipating that citizen scientists would require training to contextualize their involvement in the study and develop their research skills for meaningful involvement in different research tasks, we asked citizen scientists to complete an online form asking for their needs and preferences for attending research skill development training.

Ethical issues and research governance

Before the recruitment, each potential citizen scientist who showed interest in our study was provided with a copy of the Citizen Scientists Information Sheet (CSIS) and an Expression of Interest (EoI) form by email. The CSIS provided information about the research study and research project, who we were looking for, research tasks to be involved in, and the duration of involvement in the study (about 24 months). The CSIS also made clear that any citizen scientist could leave the study at any time without giving a reason, work on an unpaid and voluntary basis in their free time, decide how much time they wanted to devote to the study, and choose which research activities they wanted to be involved in. The CSIS also mentioned that (although participation as a citizen scientist was voluntary and without any salary), reimbursement of expenses, attendance at any meetings, and covering any caring costs were possible according to the procedures of our organization and research center. The CSIS also provided the contact details of a designated researcher to ask questions, if any, and seek more information about the study and the project. The potential citizen scientists were asked to complete a consent form and declare any conflicts of interest in the study. They were informed that their data would be stored securely on a password-protected laptop and would be deidentified and aggregated before being reported in research project reports and research papers.

We also informed potential citizen scientists that they would have no access to research participants who will be participating in the clinical experiment, including clinical investigations and the 12-week lifestyle and weight loss intervention program. However, they were informed that they would have access to de-identified and aggregated data collected from the participants of the clinical experiment, and they could take part in co-analyzing these de-identified and aggregated data in collaboration with the professional scientific researchers. They would work remotely using online conferencing and meeting tools such as Microsoft Teams® (Redmond, USA) and Zoom® (California, US). Because of the COVID-19 pandemic, no face-to-face or in-person meetings were planned, and all communication and meetings were conducted online.

## Results

Recruitment of citizen scientists

**Figure 2:**
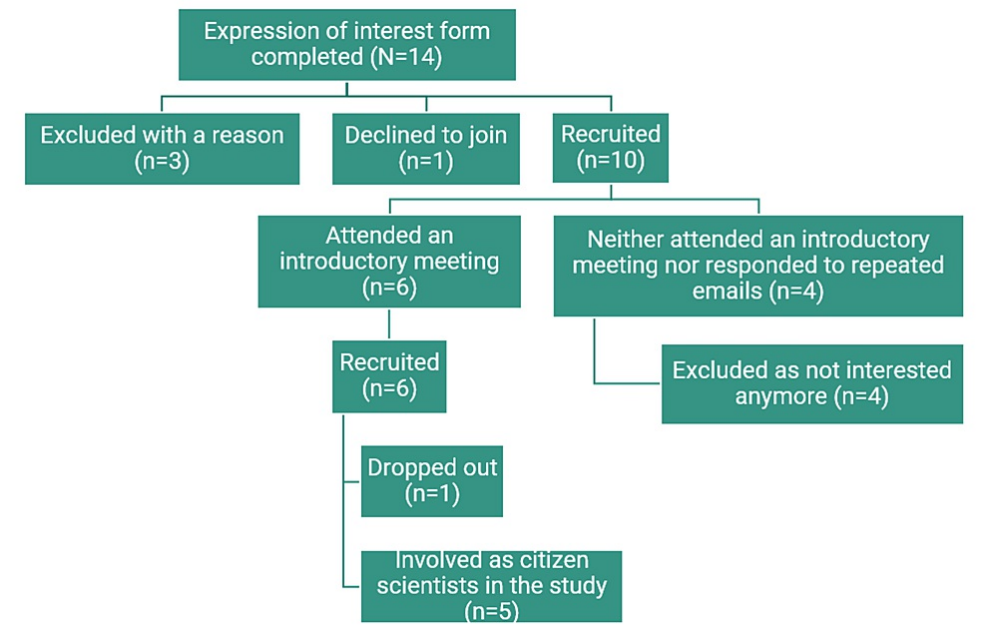
Flowchart of citizen scientists’ recruitment

Figure 2 shows the flow chart showing members of the public who were interested in, were excluded, dropped out, and were finally included as citizen scientists in the study. In response to our calls for citizen scientists, 14 members of the public expressed their interest in being involved in the study and completed EoI forms. Two of them were from countries outside Europe, i.e., one each from Australia and India; therefore, we declined them due to logistical reasons. Another person was also declined because this person was a professional researcher, had several years' research experience, and was affiliated with a higher education institution in another European country. One member of the public wanted to be paid for working on research tasks but opted out when told that their involvement would be unpaid and voluntary, except for the reimbursement of the care costs, if any, according to our institutional reimbursement policy. Thus, 10 of 14 (71%) applicants were invited to join as citizen scientists in our study and attend an online introductory meeting. However, only six of them (60%) attended the introductory meeting. We repeatedly contacted the remaining four members of the public who had completed the EoI forms by email, but they did not respond. They were, therefore, considered not interested in the study anymore; hence, they were excluded. Finally, six members of the public were recruited as citizen scientists in the study (Figure 2).

Citizen scientists' socio-demographic characteristics

Table 1 presents the socio-demographic characteristics of the citizen scientists involved in our study. Most of the citizen scientists were female, more than 50 years old, educated at postgraduate level, and either retired or not in formal employment. Most of them had caring responsibilities and had experience in research while not being formal researchers. The most common routes of recruitment and hearing about our citizen science study were targeted emails and online calls for citizen scientists on the NIHR Oxford BRC website and the STEP CHANGE project website (Table 1).

**Table 1:** Socio-demographic characteristics of citizen scientists (n=6) PhD: doctor of philosophy, CSI: citizen science initiative, PPI: patient and public involvement

Socio-demographic characteristics	Category	Frequency
Gender	Female	83%
	Male	17%
Age (Years)	Mean (Standard Deviation)	55.7 (13.9)
	Median	50.5
Ethnicity	White	50%
	Asian	33%
	Mixed	17%
Highest education level	Postgraduate diploma	17 %
	Master’s degree	66%
	PhD	17%
Caring responsibilities	Yes	50%
	No	33%
	No response	17%
Employment status	Retired	33%
	Employed	17%
	Voluntary work	17%
	Unpaid family carer	17%
	Not in employment	17%
Previous research experience	Yes	50%
	Minimal	33%
	None	17%
Hearing about the CSI (Recruitment route)	Presentation to a PPI group	17%
	Targeted email to PPI groups	50%
	Online call on the project and BRC websites	33%

Retention of citizen scientists

The retention rate of citizen scientists was 83%, while the dropout rate was 17%. Only one citizen scientist dropped out of the study due to personal health reasons after about five months of involvement in the study, thus leaving five citizen scientists involved in the study. Interestingly, this led to parity between citizen scientists (n = 5) and professional researchers (n = 5) involved in the study.

Incentives for citizen scientists

**Figure 3:**
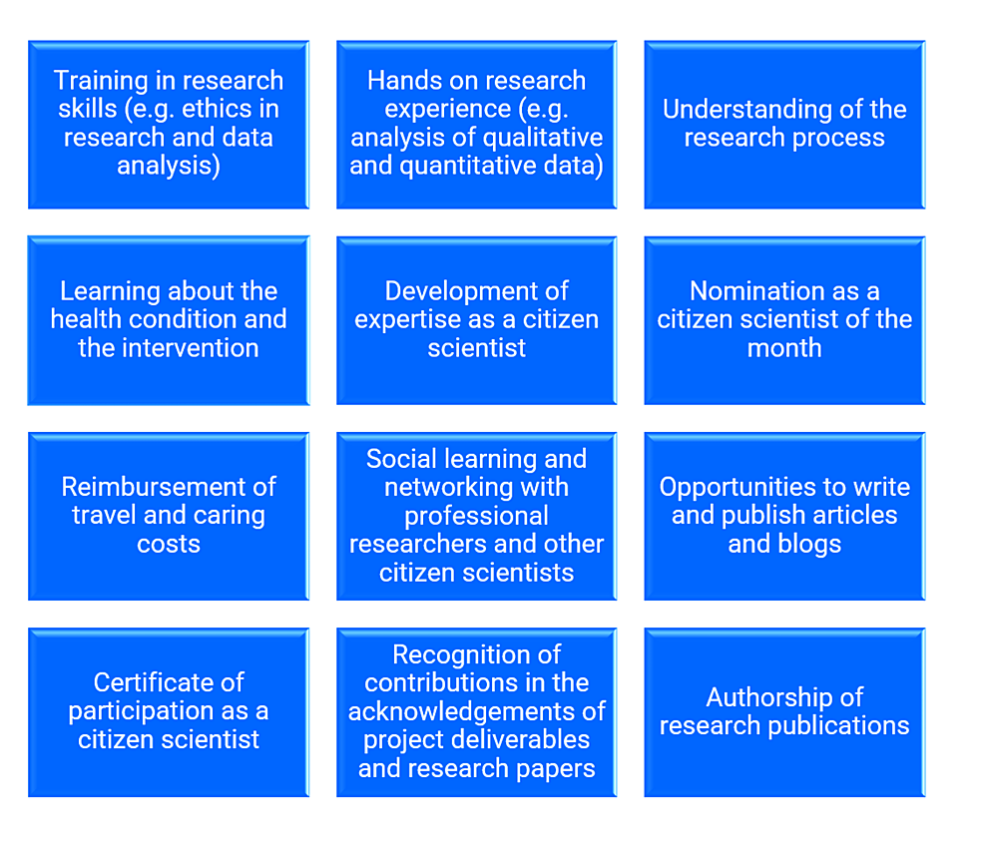
Incentives for citizen scientists

We offered different incentives to citizen scientists [21], and they capitalized on most of the incentives (Figure 3). For example, the reimbursement of care costs, training in research ethics and data analysis, and nomination for the ‘citizen scientist of the month’ (thus publication of their profiles on the project website and their dissemination through social media). Two citizen scientists wrote perspectives in articles that were published on the website as well as in the six-monthly newsletter of the STEP CHANGE project. 

Training of citizen scientists

Citizen scientists were interested in attending training to develop their skills for different research tasks and activities, including the development and review of data collection tools, the collection and analysis of research data, the interpretation and synthesis of research findings, the dissemination of research findings; and the drafting of research papers. They preferred online training in a group, either in the morning or afternoon, led by an instructor. The most convenient days for attending training were Wednesdays and Fridays. They had no preference for the duration of a training session.

Research skills development training was provided at a time and day of the week that was convenient to most of them. All training sessions (n = 11) were online, interactive, and led by an instructor who was a professional researcher with several years of experience in health and biomedical research and affiliated with the NIHR Oxford Biomedical Research Centre. Training was mostly provided to a group of citizen scientists (seven sessions), and the duration of each group training session was about two hours. Citizen scientists who could not attend a group training session were offered one-on-one training in hour-long sessions (four sessions in total). The main areas of training were ethics in research and qualitative and quantitative data analysis. They were provided opportunities to apply their research skills through hands-on practice, as described in the following section.

Roles and contributions of citizen scientists

**Figure 4:**
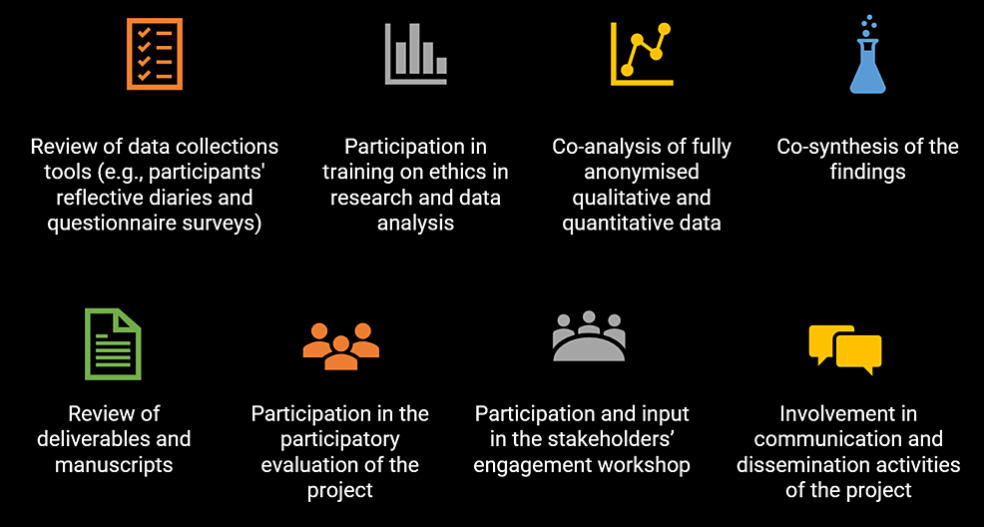
Citizen scientists’ research activities

Citizen scientists were involved as co-researchers alongside professional researchers in the study. They contributed to various research activities (Figure 4). They reviewed questionnaires for data collection from patients participating in the experiment. They analyzed deidentified quantitative data using descriptive statistics and created visualizations, as well as deidentified qualitative data through thematic analysis and created word clouds, which were reported in a project deliverable. They also wrote perspective articles and blogs, which were published on the STEP CHANGE project website and in the project newsletter. Citizen scientists reviewed and made constructive comments on two project deliverables and a research paper. Their contributions were duly acknowledged, either as a group or by name, where appropriate, in the project outputs. Co-authoring of a research paper with citizen scientists is in the pipeline. One of the citizen scientists was a member of the participatory evaluation team for the project and took part in the evaluation activities. Citizen scientists also participated in the final participatory evaluation workshop, which was held in person, as well as in a stakeholders’ engagement workshop, which was held online.

## Discussion

In this paper, we have described our methods and approaches for the successful recruitment, retention, and training of citizen scientists who were involved in an experimental medicine study on NAFLD involving a lifestyle and weight loss intervention [21]. Our findings show that women who are white, older than 50 years, and not in employment are more likely to become citizen scientists in translational medicine research in a UK setting. We also found that recruiting citizen scientists of diverse socio-demographic backgrounds is possible, but it requires the use of various methods of outreach to potential citizen scientists. Our study has also revealed that a greater number of members of the public show interest in becoming citizen scientists, but only a small proportion of them become involved as citizen scientists in the research study. We also found that dropout among citizen scientists is possible, but the rate is not very high.

Findings of our study revealed that citizen scientists take a greater interest in research skills development training; however, the training delivery and format need to be according to their schedules. Moreover, we found that the training content needs to cover basic concepts with examples explained in simple and easy language. Our study also showed that citizen scientists are interested in being involved in different research tasks but that they need support and encouragement. In addition, we found that offering some incentives could enhance citizen scientists’ interest, motivation, and involvement in the research study. These findings are discussed below.

Recruitment of citizen scientists

To recruit citizen scientists, we used various channels, of which targeted emails and placing online calls proved to be the most successful. Although online calls and social media messages appear to be promising channels to attract members of the public, the issue is that their global scope may result in people applying from different countries, which may pose logistical problems. For example, we received interest from a member of the public from Australia, which has an estimated nine-hour time difference compared to the UK time. Such a difference in time zones can become problematic when arranging meetings and training as a group, causing inconvenience for both the citizen scientists and the scientific researchers involved in the research study. To avoid these issues, we suggest the use of outreach and recruitment methods that are convenient and appropriate for the study setting, and recruitment calls should specify the scope of the study and criteria for the potential citizen scientists to avoid any inconvenience to both the potential citizen scientists and the recruiting researchers.

Retention of citizen scientists

Retaining citizen scientists [17] and preventing them from dropping out of research studies [14] are critical issues in citizen science research. In our study, we were successful in retaining the majority of recruited citizen scientists (83%); nonetheless, about 17% of citizen scientists dropped out of our study, which was not surprising. We found that once citizen scientists had joined the study, devoted their time to various research activities, and received research skills training, they were more likely to stay involved in the research study. Another possible explanation for citizen scientists’ continued involvement in our research study could be that they see some incentives offered to them [21] and availed of by themselves as beneficial. For example, some citizen scientists in our study were nominated as citizen scientists of the month, and a few of them wrote articles that were published on the project websites as well as in the six-monthly newsletter of the project, and they were disseminated through social media. All these incentives could have had a positive influence on them, thus inclining them to continue their involvement in our study. However, we do not exactly know what prompted them to continue their involvement over several months in the research study. This is because all our citizen scientists have not yet benefited from all the incentives that we have offered them [21], as the project is ongoing and some of the incentives, such as the authorship of research papers, are in the pipeline.

Training of citizen scientists

Citizen scientists could be interested in participating in various research tasks, such as data collection and analysis [7]. However, they might not have expertise in undertaking these tasks; hence, providing them training to develop their skills in various research tasks is important [17,19]. In our study, we provided training to citizen scientists on different topics, such as ethics in research and quantitative and qualitative data analysis. Training provided was well received by citizen scientists, and it has had a very positive impact, which is evident from the articles, one on ethics and one on data analysis, written by two citizen scientists involved in the study. However, challenges in providing training to citizen scientists include planning training sessions that are convenient to citizen scientists, ensuring the training delivery schedule and format are flexible, and sometimes making sure training on a one-to-one basis is available to accommodate citizen scientists’ individual schedules and commitments. This is imperative because they are volunteers, and they might have other more important commitments, such as caring responsibilities. Moreover, in developing and delivering training to citizen scientists, the scientific researchers and training providers need to use plain language to explain often complex theoretical concepts and statistical terms and formulas in clear and simple ways, rather than using scientific terminologies and jargon. The training providers need to be flexible, patient, and available to respond to citizen scientists’ questions and queries. In addition, the training providers need to be prepared to help trainee citizen scientists go through hands-on experiences, for example, the analysis of various types of data in a group as well as one-on-one sessions.

Roles and contributions of citizen scientists

Our findings show that citizen scientists could successfully contribute to different research activities and tasks [1], starting with the review and development of data collection tools, data collection and analysis, and the synthesis of the findings. Our study also showed that citizen scientists can be involved in research project participatory evaluation activities and stakeholders’ engagement events.

Limitations of the study

Our study has a few limitations. First, we tried a variety of recruitment methods, including targeted emails, online calls, and social media messaging, but we were unable to draw in many citizen scientists. This could be because experimental medicine research is a highly regulated and specialized sector. Second, the findings of our study could not be generalized since we used convenience sampling instead of random sampling and involved an extremely small sample of citizen scientists. Third, we do not know the impact of research skills development training on our citizen scientists, which could be assessed through the research project evaluation process. Additionally, the COVID-19 pandemic prevented us from setting up in-person group activities like training or meetings, which would have been a better way to foster group dynamics, learn from professional researchers, interact with like-minded citizen scientists, and create social and research networks.

## Conclusions

Our study shows that citizen science approaches can be successfully applied in translational medicine research, and citizen scientists can participate in different research activities, contribute to the co-creation of research outputs, and thereby co-produce scientific knowledge. Citizen scientists voluntarily and actively participate and invest their time and efforts in co-conducting various research tasks in collaboration with scientific and clinical researchers. However, they need support and training to develop their research skills and expertise. The training provided to them needs to accommodate their individual schedules, and it should be flexible, simple, and comprehensive. Recruiting citizen scientists is challenging; however, applying various channels of recruitment could help in addressing this issue. There is a chance that some of them could opt to drop out of the research study or project; however, the dropout rate could be limited if they see their time and involvement in the study as meaningful and worthwhile of their time and effort, and if they see that they are contributing to a study whose outcomes could be beneficial not only for them but also for others. Moreover, incentives provided to the citizen scientists could also help retain them over the course of the study.

Nevertheless, recruiting, retaining, and training citizen scientists in translational medicine research takes time and requires resources and planning. Scientific researchers must consider these issues while designing citizen science studies and projects in the fields of translational medicine and biomedical research.
